# Pyoderma Gangrenosum Following Cesarean Section: A Case Report With Individualized Management

**DOI:** 10.1155/crog/2579304

**Published:** 2026-06-26

**Authors:** Zepeng Zheng, Shilian Xu, Xiaomiao Zeng, Xiaojun Chen, Xiaoli Liu, Shuning Zhang, Shuai Fu

**Affiliations:** ^1^ Department of Obstetrical, Shenshan Medical Central, Memorial Hospital of Sun Yat-sen University, Shanwei, China; ^2^ The Wound and Ostomy Care Clini, Shenshan Medical Central, Memorial Hospital of Sun Yat-sen University, Shanwei, China

**Keywords:** cesarean section, postoperative complication, postpartum, pyoderma gangrenosum

## Abstract

**Introduction:**

Pyoderma gangrenosum (PG) is a rare neutrophilic dermatosis characterized by rapidly progressive necrotizing skin ulcers. PG following cesarean section (CS) is extremely rare and frequently misdiagnosed as surgical site infection, leading to inappropriate debridement and disease exacerbation. We report a case of severe postcesarean PG successfully managed with early immunosuppressive therapy.

**Case Presentation:**

A 24‐year‐old primiparous woman at 29 + 4 weeks gestation underwent emergency CS due to umbilical cord prolapse. On postoperative day (POD) 7, she developed painful ulcers with undermined violaceous borders around the incision. Despite broad‐spectrum antibiotics, lesions progressed rapidly. Pathergy occurred following surgical debridement on POD 9 (wound enlarged to 21.5 × 11.0 cm). PG was diagnosed on POD 11 based on clinical features, sterile cultures, and neutrophilic infiltrate on histopathology. Treatment included methylprednisolone pulse therapy (500 mg intravenously daily for 3 days), followed by tapering to 60 mg/day with concurrent initiation of cyclosporine (75 mg oral twice daily) and etanercept (25 mg subcutaneously twice weekly). The patient was discharged on POD 30. Complete epithelialization was achieved by the 4th postoperative month without recurrence or adverse drug reactions.

**Discussion:**

This case highlights the diagnostic challenges of postcesarean PG, which requires high clinical suspicion to distinguish from infectious complications. Triple immunosuppressive therapy (high‐dose corticosteroids, calcineurin inhibitor, and TNF‐*α* antagonist) represents an effective strategy for severe, rapidly progressive disease, allowing rapid disease control while facilitating corticosteroid tapering. Long‐term follow‐up confirmed sustained remission despite extensive initial ulceration.

## 1. Introduction

Pyoderma gangrenosum (PG) is a rare and severe neutrophilic dermatosis characterized by rapidly progressing, painful skin ulcers with undermined violaceous borders [1]. PG can occur spontaneously or following minor trauma, surgery, or infection [2]. The exact etiology of PG remains unclear, but it is often associated with systemic inflammatory conditions such as inflammatory bowel disease, rheumatological disorders, and hematological malignancies [1].The incidence of PG is approximately 3–10 cases per million population [3]. Women are more frequently affected than men [3].

In the context of pregnancy and obstetrics, PG is particularly challenging due to the altered immune state of pregnant women [4]. The pathogenesis is thought to be related to the progressive neutrophilia and changes in cytokine balance that occur during pregnancy [5–7]. Specifically, the shift towards Th1 and Th17 cytokines at the end of pregnancy may predispose certain individuals to PG. The diagnosis is often delayed due to its rarity, the initial presentation mimicking other common skin conditions and the absence of specific diagnostic markers, which may occur devastating consequences [8]. Conservative management involves high‐dose systemic corticosteroids, cyclosporine, local wound care, and other immunosuppressants [9].

To our knowledge, we report a severe case of PG after cesarean delivery. This case report aims to highlight the diagnostic and therapeutic challenges of PG following cesarean section (CS) and to review the relevant literature on this topic.

## 2. Case Presentation

A primiparous woman (24 years old) was admitted to our hospital at 29 + 4 gestational weeks on September 17, 2024, because of preterm prelabor rupture of membranes. Her obstetric history included a second‐trimester fetal loss at 24 weeks of gestation. Emergency CS was performed due to umbilical cord prolapse. A female infant was delivered vigorously (weight, 1.19 kg; Apgar score: 8 at 1 min, 10 at 5 min, 10 at 10 min). The intraoperative course was uneventful.

**Figure 1:**
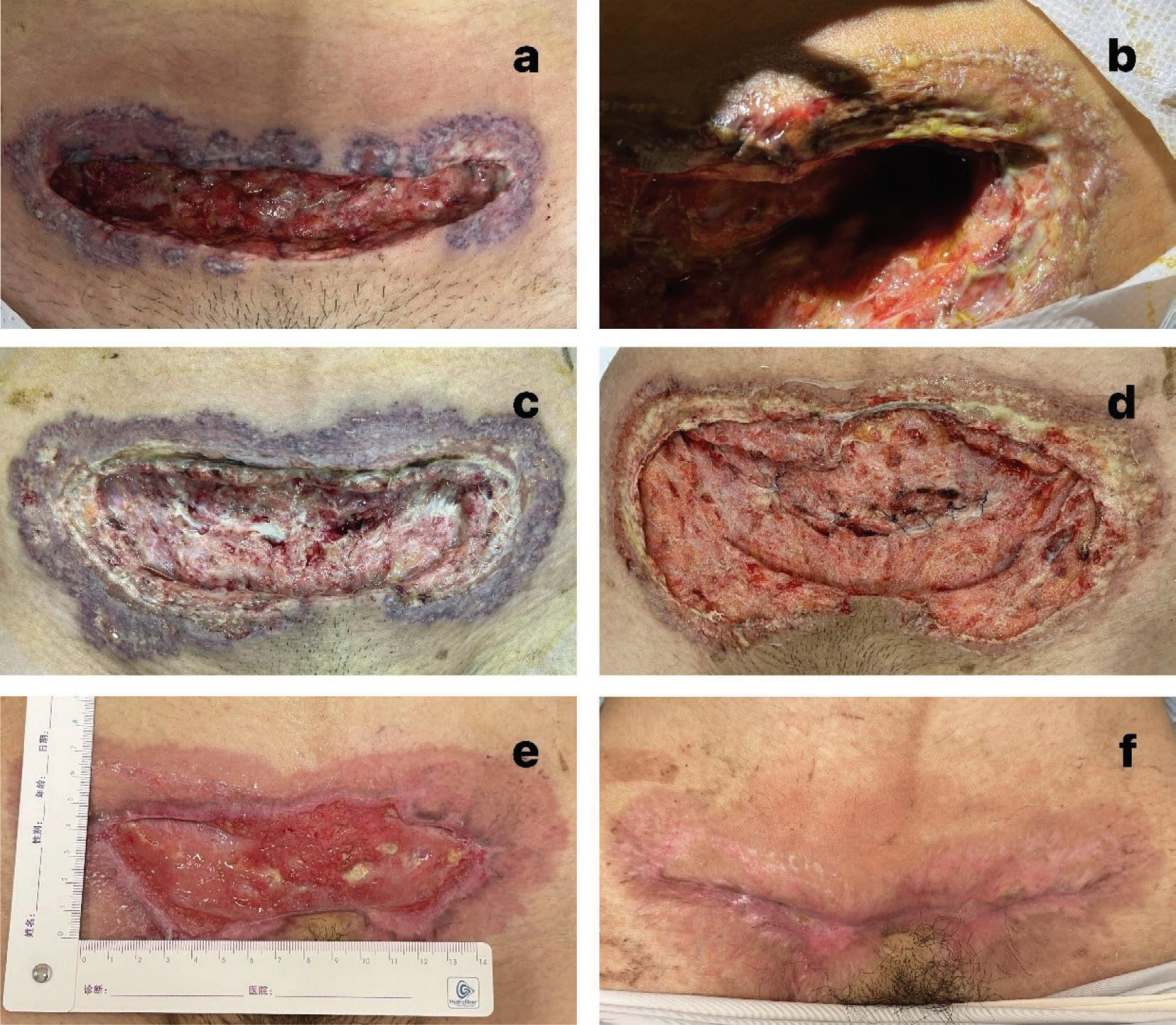
(a) POD 7, the lesion had evolved into a reddish purple, slightly elevated area with an undermined margin and was associated with severe pain. (b) POD 8, the skin around the incision developed new blisters. (c) POD 9, the diffuse purplish erythema surrounding the wound had worsened and expanded further with the development of ulcers. (d) POD 14, during the third day of systemic corticosteroid therapy, the wound exhibited no further expansion. (e) On the 2nd month postcesarean section. (f) On the 4th month postcesarean section, the wound had completely healed.

**Figure 2:**
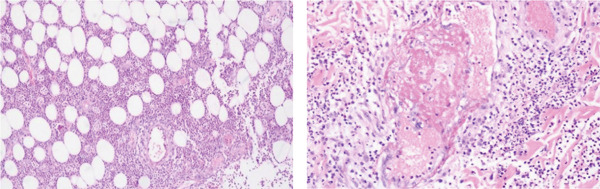
Hematoxylin section (10×) reveals marked neutrophil infiltration (left). Hematoxylin section (20×) demonstrates intravascular thrombosis (right).

On postoperative day (POD) 1, the patient developed fever (maximum temperature, 39.4°C). Laboratory investigations revealed leukocytosis (WBC: 22.85 × 10^9^/L with neutrophilia) and elevated C‐reactive protein (CRP: 26.37 mg/L) (Table 1). Puerperal infection was suspected, and antibiotic therapy was upgraded from cefuroxime to piperacillin‐tazobactam (4.5 g every 8 h). On POD 7, widespread bullous lesions with yellowish exudates and deep ulcerations with undermined violaceous borders developed around the surgical incision following removal of skin sutures (Figure 1a). Broad‐spectrum antibiotics including cefoperazone‐sulbactam, tigecycline, linezolid, meropenem, vancomycin, and metronidazole were administered sequentially without clinical improvement. Blood, wound exudate, vaginal, and urine cultures were all negative, as was next‐generation sequencing (NGS) of blood and wound specimens. On POD 8, CRP increased to 237.9 mg/L and WBC rose to 33.9 × 10^9^/L (Table 1), indicating disease progression despite antimicrobial therapy. On POD 9, surgical debridement was performed under general anesthesia, resulting in wound enlargement to 21.5 × 11.0 cm (Figure 1d). Negative pressure wound therapy (NPWT) was applied concurrently from POD 9 to 15. Histopathologic examination on POD 9 revealed dense neutrophilic infiltrate, acute inflammation, abscess formation, ulceration with hemorrhage and necrosis, and thrombotic vascular changes (Figure 2).

**Table 1:** Trends in the patient′s body temperature and infection indicators.

Indicator	9/18 Day 1	9/20 Day 3	9/22 Day 5	9/25 Day 8	9/26 Day 9	9/28 Day 11	9/29 Day 12	10/09 Day 22	10/18 Day 31
Temperature (°C)	39.4	39.5	41.0	40.3	40.6	38.8	36.4	36.8	36.4
WBC (3.5 ~ 9.5 × 10^9^/L)	22.9	24.1	19.3	34.0	30.0	19.8	16.5	14.1	12.6
Neutrophil (1.8 ~ 6.3 × 10^9^/L)	21.3	21.5	17.1	31.2	28.3	16.9	15.2	11.7	7.6
NEUT % (40%~70%)	93.2	89.4	88.6	91.8	91.8	88.6	92.3	82.7	60.3
CRP (0~6 mg/L)	26.4	—	110.0	238.0	214.0	164.8	138.0	3.6	1.9

Abbreviations: WBC, white blood cell count; NEUT %, neutrophil ratio; CRP, C‐reactive protein.

On POD 11, PG was diagnosed following multidisciplinary consultation. Intravenous methylprednisolone (500 mg/day) was initiated, eliciting a dramatic response within hours. The patient defervesced, and values of laboratory indicators of inflammation (WBC count of 19.7 × 10^9^/L and CRP level of 164 mg/L) gradually returned to normal (12.6 × 10^9^/L and 1.9 mg/L, respectively). On POD 15, methylprednisolone was tapered to 60 mg/day, with concurrent initiation of cyclosporine (75 mg oral twice daily) and recombinant human tumor necrosis factor Receptor‐II: Fc fusion protein (etanercept, Enbrel) (25 mg subcutaneously twice weekly) as steroid‐sparing agents.

The patient was discharged on POD 30 and followed up at the Wound and Ostomy Care Clinic. Postdischarge, the patient was maintained on triple therapy (prednisolone 20 mg daily, cyclosporine 75 mg twice daily, and etanercept 25 mg twice weekly), with all agents gradually tapered over the following 4 months. Outpatient management included conservative wound debridement, silver sulfadiazine‐impregnated lipid hydrocolloid dressings, and human epidermal growth factor application. Complete epithelialization was achieved by the 4th postoperative month (Figure 1f).

## 3. Discussion

PG is a rare neutrophilic dermatosis that poses significant diagnostic and therapeutic challenges as a postoperative complication following CS. With few cases documented in the global literature to date [10–19] (Table 2), postcesarean PG typically manifests within the first postoperative week (median onset: POD 4–5; range: POD 1–23), presenting initially with nonspecific inflammatory signs—including erythema, marked edema, disproportionate pain, and fever—that rapidly progress to painful ulcers with undermined violaceous borders and necrotic bases. The diagnosis remains predominantly clinical, necessitating systematic exclusion of infectious etiologies, as characteristic histological findings of dense neutrophilic infiltrates without definitive vasculitis are nonspecific and may be absent in early lesions. Crucially, wound and blood cultures are consistently sterile despite purulent appearances, a feature that, combined with the pathognomonic pathergy phenomenon (paradoxical worsening following surgical debridement observed in 40% of cases), should alert clinicians to the possibility of PG when patients fail to respond to antibiotics.

**Table 2:** Summary of reported cases of pyoderma gangrenosum following cesarean section.

Author/year	Age (y)	Gestation (wk)	Onset (POD)	Prior surgery history	Associated diseases	Initial misdiagnosis	Pathergy	First‐line treatment	Adjunctive therapy	Surgical intervention	Healing time	Recurrence	Follow‐up	Outcome	Key notes
Steadman et al. 1998 [10]	32	37	1	SVD with fever episode	None	Yes	No	Methylprednisolone 1 g IV qd × 3 d	None	None	1 mo	No	NS	Healed, minimal scar	First reported case; hypogammaglobulinemia
Ronnau et al. 2000 [11]	24	Term	6	Appendectomy with wound dehiscence	Hepatitis C	Yes	Yes	Prednisolone 1.2 mg/kg/d	Cyclosporine A 2.5 mg/kg/d	None	5 mo	No	NS	Complete resolution	Prior PG after appendectomy
Banga et al. 2006 [12]	32	Term	23	None	None	Yes (delayed)	No	Prednisone 65 → 100 mg/d	Topical tacrolimus 0.1%	None	6 mo	No	9 mo	Healed, fine scar	Misdiagnosed 23 d
Aydin et al. 2015 [13]	32	37	7	Prior CS wound dehiscence; IV site dehiscence	None	Yes	Yes	Prednisolone 80 → 40 mg/d + Azathioprine 100 mg/d	Minocycline	VAC → STSG (d 72)	3 mo	No	36 mo	Complete healing	Steroid side effects
Nonaka et al. 2016 [14]	39	36	5	4 SA, 1 SVD, 1 prior CS	Possible APS	Yes	No	Prednisolone 60 mg/d + Minocycline 200 mg/d	Topical steroids	None	3 mo	No	10 mo	Near‐complete epithelialization	Concurrent placenta accreta
Satoh et al. 2018 [15]	27	33	4	Appendectomy with PG (10 y ago)	None	Yes	Yes	Prednisolone 40 mg/d	None	None	2 mo	No	NS	Epithelialized	10‐y interval recurrence
Shen et al. 2019 [16]	32	>28 (preterm)	2	None	Chorioamnionitis	Yes	No	Methylprednisolone 80 mg IV + pulse 250 mg	Cyclosporine A, IVIG, HBO	VSD, skin graft	3 mo	No	12 mo	Healed	Extensive ulcer (25 × 15 cm)
Foessleitner et al. 2019 [17]	34	30 (preterm)	4	None	None	Yes	Yes	Prednisolone 500 mg IV → Methylprednisolone 20 mg PO bid	Topical steroids	2 debridements (worsened)	1 mo	Yes (POD 64)	6 mo	Healed, recurrence	Depression from steroids
Gunduz et al. 2020 [18]	32	Term	3	None	None	Yes	No	IVIG 0.4 g/kg/d × 5 d monthly	Methylprednisolone pulse	Skin graft attempted (failed)	3 mo	No	12 mo	Complete healing, atrophic scars	Refractory case
Rao et al. 2022 [19]	Early 30s	Term	<1	None	None	Yes	No	Prednisone + Cyclosporine	NPWT	STSG (3 wk)	3 wk	No	48 mo	Complete healing	Rapid progression

*Note:* Healing time refers to complete epithelialization or wound closure. Follow‐up indicates the duration of posttreatment observation without recurrence. Pathergy refers to worsening of lesion after surgical trauma.

Abbreviations: APS, antiphospholipid syndrome; bid, twice daily; CS, cesarean section; d, days; HBO, hyperbaric oxygen; IV, intravenous; IVIG, intravenous immunoglobulin; mo, months; NPWT, negative pressure wound therapy; NS, not specified; PO, per os (oral); POD, postoperative day; qd, once daily; SA, spontaneous abortion; STSG, split‐thickness skin graft; SVD, spontaneous vaginal delivery; VAC, vacuum‐assisted closure; VSD, vacuum sealing drainage; wk, weeks; y, years.

Differentiation from surgical site infection and necrotizing fasciitis is imperative [20, 21]; unlike these infectious complications, postcesarean PG demonstrates disproportionate pain, rapid progression despite adequate antimicrobial therapy, and the absence of crepitus or fascial involvement on imaging. Notably, approximately 90% of reported cases were initially misdiagnosed (Table 2), with inappropriate debridement frequently exacerbating tissue destruction and delaying definitive immunosuppressive therapy. In this case, we performed two debridements, which led to an increasing extent of the wound (Figure 1d). So, heightened clinical suspicion, early dermatologic consultation, and multidisciplinary collaboration among obstetricians, dermatologists, and surgeons are essential to prevent iatrogenic worsening and optimize patient outcomes.

Treatment of PG typically begins with systemic corticosteroids [4]. According to the Japanese Dermatological Association (JDA) 2022 guidelines, the standard initial dose of systemic corticosteroids for PG is 0.5 mg/kg/day of prednisolone, with a maximum of 1 mg/kg/day (capped at 75 mg/day) reserved for severe ulcerative cases [1]. Cyclosporine (3–5 mg/kg/day) is an immunosuppressive drug also used as a first‐line choice [1]. However, drug dosing is largely empirical owing to limited evidence‐based guidance, and a study demonstrated that almost half of the enrolled patients receiving oral prednisolone (0.75 mg/kg/day) or cyclosporine (4 mg/kg/day) did not obtain healing of PG ulcers at 6 months, but almost a third of patients in both treatment groups developed a disease recurrence [22]. On the other hand, Pulse therapy with 1000 mg of intravenous methylprednisolone for 3–5 days, followed by oral corticosteroids, may have a faster onset of action and may also help taper oral corticosteroids [9, 23]. In this case, high‐dose pulse therapy of intravenous methylprednisolone (500 mg/day for three consecutive days) was initiated at first, given the extensive wound surface area (> 200 cm^2^), ongoing progressive infiltration, and significant pain. As anticipated, rapid alleviation of pain and pyrexia was observed, indicating a favorable treatment response. On Day 4 following pulse therapy, methylprednisolone was tapered to 60 mg/day and subsequently reduced in four steps to 20 mg/day during hospitalization. Complete ulcer healing was achieved by the 4th month post‐CS (Figure 1f), with sustained remission and no documented adverse drug reactions.

Alternative options include TNF‐*α* inhibitors, such as etanercept [24]. In this case, to facilitate corticosteroid tapering while mitigating relapse risk, etanercept was selected (25 mg subcutaneously twice weekly). Although etanercept has demonstrated a somewhat lower complete response rate (61%) compared with monoclonal antibodies such as adalimumab (75%) in a semisystematic review of TNF‐*α* inhibitors, its favorable safety profile as a TNF‐*α* receptor fusion protein renders it particularly suitable for postcesarean PG patients [25]. Nevertheless, Maronese et al. [9] emphasized that TNF‐*α* inhibitor therapy in postpartum women necessitates careful risk‐benefit assessment regarding lactation.

In some instances, intravenous immunoglobulin has proven to be a valuable option [18], particularly when first‐line treatments fail or cause significant complications. Surgical treatment is controversial in PG; a study has shown that split thickness skin grafting (STSG) combined with NPWT is an effective method for treating PG, significantly improving skin graft survival rates [26]. There are also two cases of PG after CS where NPWT was successfully used [13, 16]. In this case, we considered using STSG on the 2nd month after CS (Figure 1e), but the patient did not accept it.

Wound care is also indispensable. It has been reported that the mortality rate of PG is three times higher compared with the general population [26]. In this case, conservative sharp debridement was used to care for the wound until complete healing. During the care period, silver sulfadiazine‐impregnated lipid hydrocolloid dressing and human epidermal growth factor were successively used to promote wound healing. No secondary infection occurred.

Recurrence of PG is common following subsequent surgical procedures or local trauma, including CS [13, 15, 27]. When surgery is unavoidable, prophylactic perioperative immunosuppressive therapy should be considered; however, this approach is challenging due to potential adverse effects and the complexity of dosing to ensure safety for both the mother and fetus [4]. Ohmaru‐Nakanishi et al. [28] reported a 28‐year‐old woman who developed perineal PG during pregnancy (at 36 weeks and 4 days of gestation), underwent cesarean delivery at 38 weeks of gestation while taking oral prednisolone (15 mg/day), and the surgical incision did not develop PG during subsequent observation. This case seems to imply that it is possible to prevent the recurrence of PG at CS wounds with low‐dose corticosteroids, but we need more data to prove it.

## Author Contributions

Zepeng Zheng and Shilian Xu designed the study, analyzed the data, and drafted the manuscript, both of which have equal contribution. Shuai Fu and Shuning Zhang participated in the critical discussion and revision of the article. Xiaomiao Zeng, Xiaojun Chen, and Xiaoli Liu assisted in the article writing and revision.

## Funding

This research was supported by the Shanwei Provincial Science and Technology Supporting “Hundred‐Thousand‐Ten Thousand Program” Special Fund Project (Grant No. 2025B001).

## Disclosure

All authors have read and approved the final version of the manuscript. S.F. had full access to all of the data in this study and takes complete responsibility for the integrity of the data and the accuracy of the data analysis. S.F. affirms that this manuscript is an honest, accurate, and transparent account of the study being reported; that no important aspects of the study have been omitted, and that any discrepancies from the study as planned (and, if relevant, registered) have been explained.

## Ethics Statement

All the procedures of this study conformed to the 1964 Helsinki Declaration and its later amendments or similar ethical standards and passed the examination and approval of the Ethics Committee of Shenshan Medical Central, Memorial Hospital of Sun Yat‐sen University (Ethics No.: 2025‐SSKY‐020‐01). This study was also conducted with the patient′s written informed consent for the publication of clinical data and images, which included permission for photographic documentation.

## Conflicts of Interest

The authors declare no conflicts of interest.

## Data Availability

The data that support the findings of this study are available on request from the corresponding author. The data are not publicly available due to privacy or ethical restrictions.
